# ISOTEMPORAL SUBSTITUTION OF 24-HOUR MOVEMENT BEHAVIOR AND FRAILTY STATUS IN OLDER ADULTS

**DOI:** 10.1093/geroni/igad104.3041

**Published:** 2023-12-21

**Authors:** Ting-Fu Lai, Yung Liao, Ming-Chun Hsueh, Chung-Sheng Lee, Kun-Pei Lin, Ding-Cheng (Derrick) Chan, Yung-Ming Chen, Chiung-Jung Wen

**Affiliations:** National Taiwan Normal University, Taipei City, Taipei, Taiwan (Republic of China); National Taiwan Normal University, Taipei City, Taipei, Taiwan (Republic of China); University of Taipei, Taipei, Taipei, Taiwan (Republic of China); Kainan University, Taoyuan, Taoyuan, Taiwan (Republic of China); National Taiwan University Hospital, Taipei City, Taipei, Taiwan (Republic of China); National Taiwan University Hospital, Taipei City, Taipei, Taiwan (Republic of China); National Taiwan University, Taipei City, Taipei, Taiwan (Republic of China); National Taiwan University Hospital, Taipei City, Taipei, Taiwan (Republic of China)

## Abstract

**Background:**

Properly distributing twenty-four hours (24-h) movement in daily life may provide older individuals chances to prevent frailty. This study aimed to investigate the effects of replacing sedentary behavior (SB) with light physical activity (LPA) and moderate to vigorous physical activity (MVPA) on risks of pre-frailty and frailty in older adults.

**Methods:**

We recruited 196 older adults (48.5% men; 80.3 ± 7.1 years old) to wear a triaxial accelerometer on the waist. Pre-frailty and frailty were assessed by a modified version of Fried’s criteria. Isotemporal substitution analysis was performed to investigate the association between 24-h movement and risks of pre-frailty and frailty.

**Results:**

After adjusting confounding variables, the results also indicated that only reallocating 60minutes of SB per day to MVPA was less likely to have pre-frailty (OR = 0.01, 95% CI = 0.01, 0.20) and frailty (OR = 0.02, 95% CI = 0.02, 0.36). In addition, each 5-min increment in MVPA from 20 min as part of total physical activity (40 min in LPA) to replace 60 minutes of SB per day resulted in being less likely to have frailty (OR = 0.546, 95% CI= 0.301, 0.992).

**Conclusions:**

This study demonstrated that using 60-min MVPA to substitute SB daily may theoretically reduce the risk of being pre-frailty and frailty. Moreover, replacing 60-min SB daily with mixed 40-min LPA and 20-min MVPA was able to reduce frailty risks. Therefore, those findings may provide alternative approaches with acceptable intensity and feasibility when promoting the physical health of older adults.

